# Higher frailty burden in older adults with chronic constipation

**DOI:** 10.1186/s12876-021-01684-x

**Published:** 2021-03-25

**Authors:** Jihye Lim, Hyungchul Park, Heayon Lee, Eunju Lee, Danbi Lee, Hee-Won Jung, Il-Young Jang

**Affiliations:** 1 Department of Gastroenterology, Asan Medical Center, University of Ulsan College of Medicine, 88 Olympic-ro 43-gil, Songpa-gu, Seoul, Republic of Korea; 2 Department of Gastroenterology, Ilsan Paik Hospital, Inje University College of Medicine, Goyang, Republic of Korea; 3 Divison of Pulmonary, Critical Care, and Sleep Medicine, Department of Internal Medicine, Eunpyeong St. Mary’s Hospital, College of Medicine, The Catholic University of Korea, 1021 Tongil-ro, Jingwan-dong, Eunpyeong-gu, Seoul, South Korea; 4 Division of Geriatrics, Department of Internal Medicine, Asan Medical Center, University of Ulsan College of Medicine, 88 Olympic-ro 43-gil, Songpa-gu, Seoul, 05505 Republic of Korea; 5 PyeongChang Health Center and County Hospital, 11 Noseong-ro, Pyeongchang-gun, Gangwon-do Republic of Korea

**Keywords:** Aging, Bowel motility, Geriatric assessments

## Abstract

**Background:**

Despite constipation being a common clinical condition in older adults, the clinical relevance of constipation related to frailty is less studied. Hence, we aimed to investigate the association between chronic constipation (CC) and frailty in older adults.

**Methods:**

This is a cross-sectional analysis of a population-based, prospective cohort study of 1278 community-dwelling older adults in South Korea. We used the Rome criteria to identify patients with irritable bowel syndrome with predominant constipation (IBS-C) and functional constipation (FC). We investigated whether participants consistent with the criteria for IBS-C and FC had CC. Frailty was assessed using the Cardiovascular Health Study (CHS) frailty phenotype.

**Results:**

In the study population with a mean age of 75.3 ± 6.3 years, 136 (10.7%) had CC. The participants with CC were older, had higher medication burdens, and had worse physical performances compared to those without CC (All *P* < .05). By association analysis, the prevalence of CC was associated with frailty by the CHS criteria (*P* < .001). The CHS frailty score was associated with the presence of CC by the univariate logistic regression analysis and the multivariate analysis adjusted for age, sex, and multimorbidity.

**Conclusions:**

Frailty was associated with CC in community-dwelling older people, suggesting that constipation should be considered as an important geriatric syndrome in clinical practice concerning frail older adults.

**Supplementary Information:**

The online version contains supplementary material available at 10.1186/s12876-021-01684-x.

## Background

Constipation is one of the most prevalent functional bowel disorders in older adults, and its prevalence in the general population is estimated to be approximately 10–20% [1–4]. The incidence of constipation is influenced by age, diet, lifestyle, and medical conditions, while medications are a known major cause of constipation in older adults [5, 6]. Furthermore, constipation negatively affects the quality of life and is a significant driver of increasing health care costs [7, 8]. Clinically, constipation may lead to hemorrhoids, rectal prolapse, depression, and occasionally, ileus or colonic perforation [9]. Despite these adverse health effects, constipation in older adults has been overlooked and is often undertreated in routine clinical practice.

Frailty, a common clinical syndrome in older adults, is defined as a decrease in the physiological reserve with aging and an increased vulnerability to stressors [10]. Studies have reported the relationship between frailty and increased risks of future adverse health outcomes including hospitalization, disability, treatment-related harmful outcomes, and death [11–13]. Since frailty often co-exists with multimorbidity and decreased daily function, the interaction between the frailty spectrum and the underlying disease is an important issue in the clinical decision making process for older adults [14]. In this regard, frailty and constipation share many causative factors and mechanisms including decreased mobility, poor nutrition and water intake, higher medication burden, and chronic diseases.

While previous studies have suggested that constipation is associated with frailty [15], the clinical relationship between chronic constipation (CC), frailty, and common geriatric issues have not been completely understood yet [16]. Therefore, we aimed to evaluate the clinical associations between CC, frailty status, and geriatric syndromes in a cohort of community-dwelling older adults.

## Methods

### Study design and participants

In this cross-sectional study, we used the records of the Aging Study of Pyeongchang Rural Area (ASPRA), an ongoing population-based, prospective cohort study of older adults in Pyeongchang, Gangwon, South Korea, established in 2014. The baseline design and protocol of ASPRA has been described previously [17]. The inclusion criteria for participation in ASPRA were as follows: (1) age greater than 65 years; (2) registration in the National Healthcare Service; (3) ability to self-ambulate with or without walking aid; (4) residing at home; and, (5) capability to provide informed consent themselves or through a proxy. Individuals who were institutionalized, hospitalized, or bed-ridden with nursing home-level care at the time of recruitment were excluded. The characteristics of the participants in the baseline population of ASPRA were generally comparable to those of a national representative sample of the South Korean rural population [17].

The questionnaires of ASPRA included questions on bowel habits between December 1, 2018 and October 30, 2019. For this study, we used the records of participants who underwent evaluation in this period (n = 1278), except one person who had undergone enterostomy and was unable to respond to the bowel habits questionnaire. The protocol of this cohort study was approved by the Institutional Review Board of the Asan Medical Center, Seoul, Korea (2015-0673), and all the participants provided written informed consent.

### Constipation assessment and questionnaire

We defined the presence of CC as the presence of either defined irritable bowel syndrome with predominant constipation (IBS-C) or functional constipation (FC) using the Rome IV criteria [18]. Irritable bowel syndrome (IBS) was defined as the presence of recurrent abdominal pain on an average of at least 1 day per week in the last 3 months, with an onset of at least 6 months before the study, and consistent with 2 or more of the following criteria: (1) associated with defecation; (2) associated with changes in stool frequency; and, (3) associated with a change in the form (appearance) of stool. In the patients with IBS, IBS-C was defined as follows, with constipation as a predominant feature: (1) lumpy or hard stools in more than a quarter of defecations and (2) loose stools in less than a quarter of defecations. FC was defined as satisfying 3 criteria for at least 3 months with onset at least 6 months ago. People with FC had at least 2 positive findings in the questionnaire, the presence of rare loose stools without the use of laxatives, insufficient symptoms to diagnose the condition as IBS. The severity of constipation was scored (range 0–6, increasingly worse) by counting the number of positive items on the 6 questions of the Rome criteria—(1) straining during more than a quarter of defecations; (2) lumpy or hard stools in more than a quarter of defecations; (3) sensation of incomplete evacuation in more than a quarter of defecations; (4) sensation of anorectal obstruction/blockage in more than a quarter of defecations; (5) manual maneuvers to facilitate more than a quarter of defecations; and, (6) < 3 spontaneous bowel movements per week. In addition to the Rome criteria for IBS-C and FC, we also assessed general bowel habits including self-reported constipation, and the use of laxatives and anti-diarrheal drugs.

### Frailty assessment

The frailty status was evaluated by the Cardiovascular Health Study (CHS) frailty phenotype criteria, that modified and adopted from the original criteria for ASPRA study [13, 17, 19]. The CHS frailty phenotype included the following items: (1) exhaustion: feeling exhausted for almost all the time in the past week and answering yes to the question, “I felt that everything I did was an effort” or “I could not get going” [17]; (2) low activity level: lowest quintile in physical activity level according to the Korean version of the International Physical Activity Questionnaires Short Form [20, 21]; (3) slowness: usual gait speed < 0.8 m/s assessed by the 4-m walk test; (4) weakness: dominant hand grip strength < 26 kg for men and < 18 kg for women; and, (5) weight loss: unintentional weight loss of > 3 kg in the past 6 months [22]. The sum of the presence of the phenotypes represented the severity of frailty in the order of robust (0 point), prefrail (1–2 points), or frail (3–5 points).

### Other measurements

Multimorbidity was defined as the presence of 2 or more chronic diseases among hypertension, diabetes, malignancy, asthma, chronic lung disease, angina, myocardial infarction, heart failure, stroke, chronic kidney disease, and arthralgia [17]. Low cognition was defined as a score of < 24 on the Korean version of Mini-Mental State Examination [23]. Disability in activities of daily living was defined as the requirement of assistance in performing any of the following 7 activities: bathing, continence, dressing, eating, toileting, transferring, and washing the hands and face. Disability in instrumental activities of daily living was defined as the requirement of assistance in performing any of the 10 instrumental activities of daily living (household chores, food preparation, grooming, going out for a short distance, laundry, handling finances, managing medications for oneself, transportation, shopping, and using the telephone) [24]. Depressive mood was defined as a score of > 20 on the Korean version of the Center for Epidemiological Studies Depression scale [25]. Polypharmacy was identified as the use of ≥ 5 different medications regularly [17]. The risk of malnutrition was defined as a score of ≤ 11 on the Mini-Nutritional Assessment-Short Form [26].

### Statistical analysis

We used the *t* test and χ^2^ test to compare continuous variables and categorical variables, respectively, in the basic characteristics between populations with and without CC. Variables shown significant difference between populations with or without CC were used as potential confounders in following analyses. We evaluated the prevalence of frailty and its phenotype according to CC using the multivariate linear least square analysis adjusted for age, sex, multimorbidity, education level, polypharmacy, and malnutrition risk. Univariate logistic regression was used to identify the association between frailty (model 1) and CC. In the multivariate logistic regression analysis, we used covariables of age and sex in model 2, and age, sex, multimorbidity, education level, polypharmacy, and malnutrition in model 3, considering geriatric items that showed significant differences regarding the state of CC. We assessed the association between frailty status and the severity of constipation using the univariate and multivariate least square regression analysis adjusting for age, sex, multimorbidity, education level, polypharmacy, and malnutrition risk. Further, the severity scores of constipation according to the 3 groups of frailty status were compared using analysis of covariance (ANCOVA) with the covariables of age, sex, multimorbidity, education level, polypharmacy, and malnutrition risk with post-hoc tests using Bonferroni corrections. The statistical analysis was performed using Stata 15.0 (StataCorp, College Station, TX, USA) and a two-sided *P* value < 0.05 was considered statistically significant.

## Results

### Characteristics of the study participants

The mean age of the participants was 75.3 ± 6.3 years, and 756 of the 1277 participants (59.2%) were women. A quarter of the participants (25.4%) self-reported their constipation, whereas 132 (10.3%) had a history of using stool softeners or laxatives. Of the participants, 28 (2.2%) had IBS-C and 108 (8.5%) had FC, and they were considered to have CC. The participants with CC were older, had less formal education, had a higher burden of multimorbidity and polypharmacy, and were more likely to have the risk of malnutrition (Table 1). The participants with CC had lower activity level and slower gait speed compared to the people without CC. They had higher CHS frailty scale scores and accordingly higher prevalence of pre-frail and frail.

**Table 1:** Basic characteristics of the study population

	Without chronic constipation (n = 1141)	With chronic constipation (n = 136)	*P* value
Age, years	75.1 ± 6.2	77.1 ± 6.3	.001
Men	472 (41.4%)	49 (36.0%)	.231
Education level, years	6.1 ± 3.7	5.0 ± 3.2	.001
Monthly income < USD 500, n (%)	52 (4.6%)	8 (5.9%)	.490
Body mass index, kg/m^2^	25.1 ± 3.5	25.1 ± 3.6	.896
Multimorbidity	560 (49.1%)	85 (62.5%)	.003
Hypertension	692 (60.7%)	93 (68.4%)	.080
Diabetes	253 (22.2%)	40 (29.4%)	.058
Malignancy	76 (6.7%)	15 (11.0%)	.061
Myocardial infarction	52 (4.6%)	11 (8.1%)	.072
Polypharmacy	239 (21.0%)	47 (34.6%)	< .001
Cognitive dysfunction	292 (25.6%)	45 (33.1%)	.061
Depression	77 (6.8%)	12 (8.8%)	.369
Fall history in the past year	39 (3.4%)	6 (4.4%)	.552
Risk of malnutrition	410 (35.9%)	64 (47.1%)	.011
ADL disability	317 (27.8%)	42 (30.9%)	.447
IADL disability	230 (20.2%)	36 (26.5%)	.087
CHS frailty scale score	1.3 ± 1.1	1.8 ± 1.1	< .001
CHS frailty phenotype			< .001
Robust	329 (28.8%)	15 (11.0%)	
Pre-frail	653 (57.2%)	85 (62.5%)	
Frail	159 (13.9%)	36 (26.5%)	
Component of CHS frailty phenotype			
Exhaustion	145 (12.7%)	25 (18.4%)	.088
Low activity level	285 (25.0%)	58 (42.6%)	< .001
Slowness	389 (34.1%)	80 (58.8%)	< .001
Weakness	548 (48.0%)	74 (54.4%)	.188
Weight loss	66 (5.8%)	6 (4.4%)	.646
Self-reported constipation	236 (20.7%)	88 (64.7%)	< .001
Use of stool softener or laxatives	85 (7.4%)	47 (34.6%)	< .001

Values are expressed as mean ± standard deviation or frequency (%)

CHS frailty phenotype is defined according to the sum of the presence of the phenotypes; robust (0 point), prefrail (1–2 points), or frail (3–5 points)

*ADL* activities of daily living, *CHS* Cardiovascular Health Study, *IADL* instrumental activities of daily living

### Association between chronic constipation and frailty

**Fig. 1:**
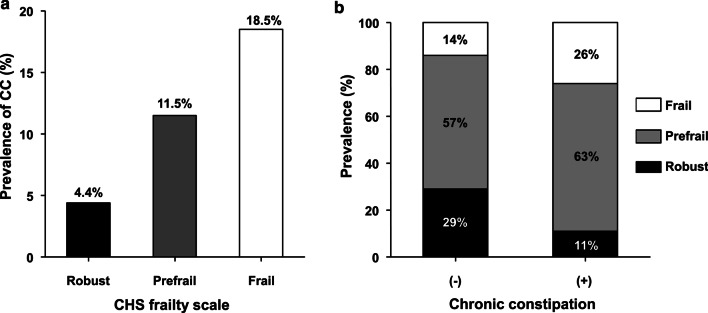
Frailty status and chronic constipation (CC) **a** Prevalence of CC according to frailty by the Cardiovascular Health Study (CHS) frailty scale **b** Prevalence of frailty status according to the presence of CC

By the CHS frailty phenotype, 344 (26.9%), 738 (57.8%), and 195 (15.3%) people were robust, prefrail and frail, respectively. The prevalence of CC was 4.4% (n = 15), 11.5% (n = 85), 18.5% (n = 36) in robust, prefrail, and frail participants, respectively (Fig. 1a). When the CHS phenotype was used as a continuous score, there was a trend of higher prevalence of CC in individuals with higher CHS score (Additional file 1: Figure S1). By the multivariate linear least square analysis adjusted for age, sex, multimorbidity, education level, malnutrition risk, and polypharmacy, the presence of CC was positively associated with the CHS scale scores (Standardized beta [B] = 0.11, *P* < 0.001). When analyzed in a categorical manner, the presence of frailty was positively associated with CC (Fig. 1b, B = 0.11, *P* = .008) by the multivariate least square regression analysis adjusted for age, sex, multimorbidity, education level, malnutrition risk, and polypharmacy.

Logistic analysis was used to evaluate the association between CC and the frailty status. When the robust status was considered as a reference, prefrail and frail statuses were associated with an increasing prevalence of CC, either by crude analysis or multivariate analysis adjusted for possible confounders (Table 2 and Additional file 2: Table S1).

**Table 2:** A logistic regression analysis showing associations between frailty status and presence of chronic constipation

	Model 1^a^		Model 2 ^a^		Model 3 ^a^	
	OR	(95% CI)	OR	(95% CI)	OR	(95% CI)
Robust (reference)	–		–		–	
Prefrail	2.86	(1.62–5.02)	2.56	(1.43–4.57)	2.44	(1.36–4.39)
Frail	4.96	(2.64–9.34)	3.93	(1.96–7.86)	3.38	(1.66–6.87)
Age	–		1.02	(0.99–1.05)	1.01	(0.98–1.05)
Sex	–		0.93	(0.63–1.38)	0.83	(0.55–1.26)
Multimorbidity	–		1.47	(1.01–2.17)	1.26	(0.84–1.88)
Education level	–		–		0.96	(0.89–1.02)
Malnutrition risk	–		–		1.17	(0.84–1.62)
Polypharmacy	–		–		1.77	(1.20–2.62)

*CI* confidence interval, *NS* not significant, *OR* odds ratio

^a^Model 1, crude model; Model 2, adjusted with age, sex, multimorbidity; Model 3, adjusted for age, sex, education level, polypharmacy, and malnutrition risk

### Severity of constipation and the frailty status

By the linear least square analysis, the severity scores of constipation were positively associated with the CHS frailty scores (B = 0.21, *P* < .001) in the crude model. Moreover, this association remained significant after adjusting for possible confounders of age, sex, multimorbidity, education level, malnutrition risk, and polypharmacy (B = 0.11, *P* = .001). In the robust, prefrail, and frail populations, the means and standard deviations of the severity score were 0.79 ± 1.28, 1.30 ± 1.65, 1.75 ± 1.83, respectively (*P* = .005 by ANCOVA, with covariables of age, sex, multimorbidity, education level, malnutrition risk, and polypharmacy). Furthermore, in the post-hoc analysis, the severity score differed significantly between the robust and prefrail groups (*P* = .019) and between the robust and frail groups (*P* = .008). However, the difference between the severity scores was not significant between the prefrail and frail groups (*P* = .322).

## Discussion

In this cross-sectional study, we found that the prevalence of CC was associated with the frailty phenotype; moreover, this association was maintained after adjusting for possible confounders. Additionally, the severity of constipation was found to be significantly associated with the increasing burden of frailty. To the best of our knowledge, this study is the first to report associations between the frailty status using widely accepted operational criteria and CC determined by the Rome criteria in community-dwelling older adults.

There are several possible shared biological mechanisms leading to the frailty phenotype and decreased bowel motility. Even though there is insufficient clinical evidence on the effects of normal aging on colonic motility, common conditions in older adults including Parkinson’s disease, diabetes, hypothyroidism, and depression are known factors that prolong colonic transit time [27]; additionally, multimorbidity is commonly associated with frailty [13]. Furthermore, older adults with these chronic diseases are frequently exposed to medications that affect colonic motility, for instance, calcium antagonists for hypertension and anticholinergic antidepressants for depression. Decreased physical activity and nutritional intake are known as factors causing physical frailty and sarcopenia [28], while adequate physical activity, and fluid and fiber intake are recommended to manage constipation [6].

We found that the prevalence of CC varied widely according to the frailty status, ranging between 4.4% in the robust population and 18.5% in the frail population, whereas the aggregated prevalence of CC was 10.7%. In addition to gastrointestinal complications of CC such as hemorrhoids, rectal prolapse, or ileus [9], recent studies have suggested that CC may increase the risks of cardiovascular diseases and chronic kidney disease due to alterations in the gut microbiota [29, 30]. Furthermore, the risk of CC increases with age, and leads to fecal impaction in older adults, which may result in geriatric syndromes of fecal incontinence, urinary retention, delirium, and falls [5, 31]. With this overarching clinical relevance of CC in older adults, clinical vigilance regarding CC while caring for people with frailty is warranted.

In our study, CC was associated with the geriatric parameters of multimorbidity, risk of malnutrition, low education, and polypharmacy in the univariate analysis and was positively associated with polypharmacy in the multivariate logistic analysis. By incorporating this evidence with the positive correlation between CHS frailty phenotype scores and the severity scores of constipation, CC may be considered as a geriatric syndrome [7], rather than an isolated disease in the older population. Specifically, polypharmacy-related issues such as potentially inappropriate medications, anticholinergic cognitive burden, and prescribing cascades [32, 33] should be assessed as factors associated with CC and should be considered while establishing therapeutic plans for CC. Moreover, as previous studies have shown that frailty can be improved by a structured multicomponent intervention [34], future studies may investigate whether CC can be improved by interventions targeting frailty and sarcopenia in older adults. Conversely, as CC is a possible reversible clinical condition in older individuals with frailty, frailty might be alleviated by optimal management targeting CC in some population. While our current study design is insufficient to further delve in these issues, future prospective study with intervention may provide more evidence on longitudinal cross-talks between CC and frailty in diverse care environments.

Our study has several limitations. First, the lack of further diagnostic tests in our data limits the accuracy of our definition of constipation, since we relied on a questionnaire in a public health cohort. The results of our study on frailty and constipation would be better interpreted as exploratory findings for hypothesis generation for in-depth future research. Second, since this study was performed in a cross-sectional manner, the causal relationship between frailty and constipation, as well as the clinical outcomes associated with CC could not be further assessed in this population. Third, as our study was conducted with older adults in a rural community in South Korea, it is unclear whether our results can be generalized to western populations, populations of older adults living in urban areas, and institutionalized populations. Further research in different populations and settings is warranted.

## Conclusions

In conclusion, our results underscore the clinical association between CC and frailty in community-dwelling older adults. As CC shares similar mechanisms with frailty, it could be better interpreted in older adults as a geriatric syndrome; patient-centered approaches that take into account physical and cognitive functions, multimorbidity, and medications are warranted.

## Supplementary Information

**Additional file 1: Figure S1.** Frailty severity and chronic constipation (CC).

**Additional file 2: Table S1.** A logistic regression analysis showing associations between Cardiovascular Health Study (CHS) frailty score and presence of chronic constipation.

## Data Availability

The raw data of the current study are not publicly available due to the protection of participants personal information but are available from the corresponding author on reasonable request.

## Abbreviations

ANCOVA: Analysis of covariance
ASPRA: Aging Study of Pyeongchang Rural Area
CC: Chronic constipation
CHS: Cardiovascular Health Study
FC: Functional constipation
IBS-C: Irritable bowel syndrome with predominant constipation
