# Supplementary material for: Higher frailty burden in older adults with chronic constipation
Source: BMC Gastroenterol. 2021 Mar 25;21:137. doi: 10.1186/s12876-021-01684-x (PMC7995705; doi:10.1186/s12876-021-01684-x)

**Figure S1.** **Frailty severity and chronic constipation (CC)**

(A) Prevalence of CC according to the sum of Cardiovascular Health Study (CHS) frailty score

(B) Prevalence of sum of CHS score according to the presence of CC


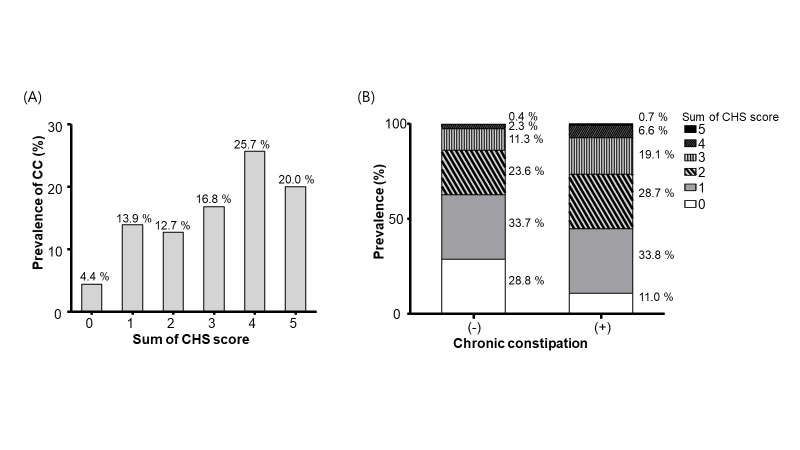

Supplement: Supplementary file 1 — Additional file 1: Figure S1. Frailty severity and chronic constipation (CC). [file 12876_2021_1684_MOESM1_ESM.docx]
