# Supplementary material for: Higher frailty burden in older adults with chronic constipation
Source: BMC Gastroenterol. 2021 Mar 25;21:137. doi: 10.1186/s12876-021-01684-x (PMC7995705; doi:10.1186/s12876-021-01684-x)
Supplement: Supplementary file 2 — Additional file 2: Table S1. A logistic regression analysis showing associations between Cardiovascular Health Study (CHS) frailty score and presence of chronic constipation. [file 12876_2021_1684_MOESM2_ESM.docx]

**Table S1. A logistic regression analysis showing associations between Cardiovascular Health Study (CHS) frailty score and presence of chronic constipation**

|  | Model 1* | | Model 2* | | Model 3* | |
| --- | --- | --- | --- | --- | --- | --- |
|  | OR | (95% CI) | OR | (95% CI) | OR | (95% CI) |
| CHS score | 1.51 | (1.29-1.76) | 1.41 | (1.18-1.68) | 1.34 | (1.11-1.61) |
| Age | - |  | 1.02 | (0.99-1.05) | 1.01 | (0.98-1.05) |
| Sex | - |  | 0.94 | (0.64-1.39) | 0.84 | (0.56-1.27) |
| Multimorbidity | - |  | 1.45 | (0.99-2.13) | 1.24 | (0.83-1.86) |
| Education level | - |  | - |  | 0.95 | (0.89-1.02) |
| Malnutrition risk | - |  | - |  | 1.14 | (0.82-1.59) |
| Polypharmacy | - |  | - |  | 1.63 | (1.08–2.45) |
